# Supplementary material for: Fast and accurate view classification of echocardiograms using deep learning
Source: NPJ Digit Med. 2018 Mar 21;1:6. doi: 10.1038/s41746-017-0013-1 (PMC6395045; doi:10.1038/s41746-017-0013-1)
Supplement: Supplementary file 1 — Supplementary Information [file 41746_2017_13_MOESM1_ESM.docx]

**Fast and accurate view classification of echocardiograms using deep learning**

Ali Madani MS, Ramy Arnaout MD DPhil, Mohammad Mofrad PhD, Rima Arnaout MD

**SUPPLEMENTARY INFORMATION**

**Supplementary Figure 1. Confidence for first and second guesses on image classification.** Box plot summarizing probabilities assigned to correct and incorrect images in the test set using the single highest probability to classify the test image. Confidence for correct answers was higher than for incorrect answers. Median, interquartile range for correct (0.999, 0.970-1.00) and incorrect (0.682, 0.525-0.867) answers.

**Supplementary Figure 2. Example native-resolution and downsampled images.** (a) Native echocardiographic images ranged from 300x400 to 768x1024 pixels in resolution, and many contained color, either as color Doppler or as different chroma maps to aid in visualization. (b) The same sample image as in (a), downsampled to the 60-by-80-pixel resolution used as input to the deep learning model.

**Supplementary Figure 1. Confidence for first and second guesses on image classification.**

**
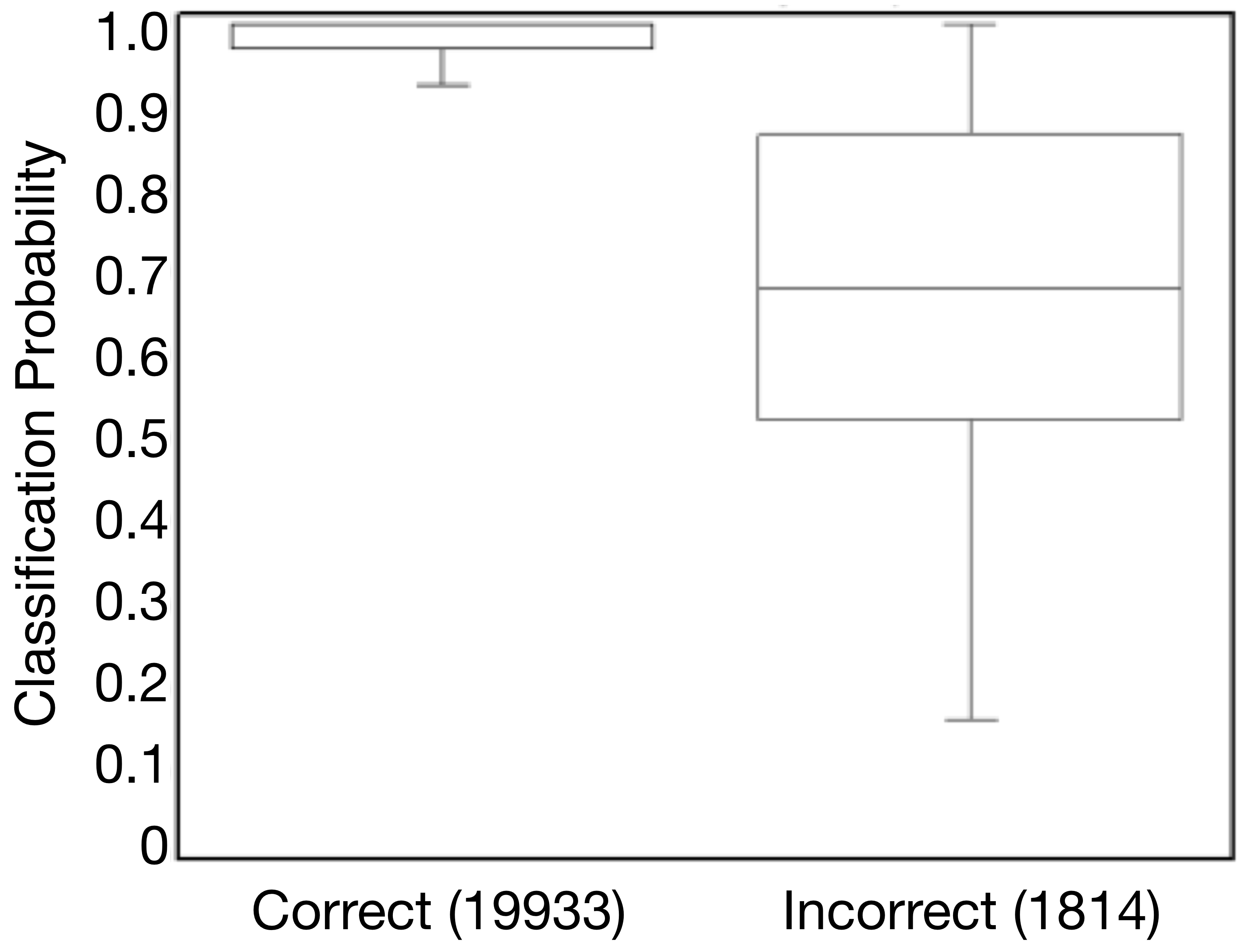
**

**Supplementary Figure 2. Example native-resolution and downsampled images.**


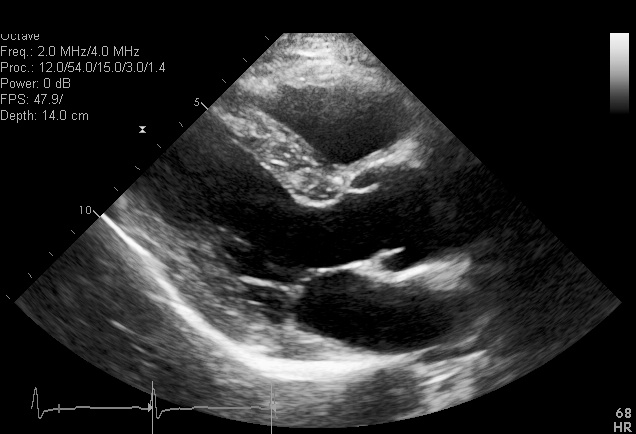
**a**


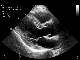
**b**
